# Immunoglobulin: unraveling its complex web in aging

**DOI:** 10.3389/fimmu.2025.1690018

**Published:** 2025-10-02

**Authors:** Qiaoli Gu, Yi Wang, Can Zhu, Xichao Zhou, Li Ni, Huan Zhao, Huilin Yang, Qin Shi

**Affiliations:** Department of Orthopedics, The First Affiliated Hospital of Soochow University, Suzhou, China

**Keywords:** immunoglobulin, aging, senescence, B cell, immunosenescence, age-related diseases

## Abstract

Aging is a complex biological phenomenon, which involved in a large number of diseases such as cancer, neurodegeneration, and cardiovascular diseases. Understanding the mechanism of aging may facilitate the development of preventive strategies of age-related diseases. Immunoglobulin (Ig) includes proteins with antibody (Ab) activity or membrane-bound proteins that share a chemically analogous structure to Ab. Ig can recognize and neutralize numerous antigens, which constitutes the main characteristic of adaptive immunity. The quantity, glycosylation and function of Ig change with advancing age. Some Ig is found to be accumulated in aged tissues and appear to be regarded as a potential marker for aging, which indicates the critical role of Ig in aging. B cells are main producers of antibodies and undergo aging-related changes, leading to increased autoimmune responses and reduced vaccine responses. The immune dysregulation of B cells is also intensively involved in the alteration of Ig. In this review, we focus on the current research findings on Ig, discuss the relation between Ig and aging, highlight the complex interplay among B cell, gut microbiota, Ig, and aging, and explore potential therapeutic strategy. We hope this review may provide an insight for investigating the regulatory mechanism of Ig in aging, as well as for evaluating the therapeutic potential in treating age-related diseases.

## 1 Introduction

Aging is a complex process that involves pronounced decrease of biological functions. It is one of the primary causes for prevalent chronic diseases such as neurodegenerative disorders, cardiovascular system diseases, and cancer, which eventually, lead to death in the world (1). Gaining insight into the mechanism underlying aging can facilitate to enhance lifespan and quality.

Many factors contribute to aging, such as genomic instability, telomere attrition, epigenetic alterations, loss of proteostasis, disabled macroautophagy, deregulated nutrient-sensing, mitochondrial dysfunction, cellular senescence, stem cell exhaustion, altered intercellular communication, chronic inflammation, and dysbiosis (2). Of which, cellular senescence has been accepted as a critical trigger of aging and received intensive investigation. Senescent cells are accumulated in tissues and organs during aging. The clearance of senescent cells has successfully alleviated aging and age-related diseases. Interestingly, accumulated immunoglobulin (Ig) is detected adjacent to senescent cells. Ig-related genes such as Igκc, Igj, and Ighg2c are also significantly increased in aged tissues. These evidences suggest that Ig-associated senescence may be a hallmark of aging (3). In other situation such as dysbiosis or chronic inflammation, Ig can also be associated with aging through gut microbiota (4). All these findings not only highlight the mechanism of aging, but also require a fresh look at the complex role of Ig in aging.

Ig is a Y-shaped glycoprotein belongs to the immunoglobulin super-family which shares the same basic structure: two heavy and two light chains linked by disulfide bonds. The alteration of specific glycans on the chains is associated with aging (5). Most importantly, Ig comprises a heterogeneous group of proteins with antibody (Ab) function and serves as the main components of the body’s humoral immunity. In vertebrates, Ig mainly exists as B cell receptor (BCR) on the surface of B cells or as Ab secreted into extracellular fluid. B cells experience immunosenescence during the process of aging and induce the dysregulation of Ig. Apart from lymphoid cells, non-lymphoid lineage and cancer cells can also express Ig (6). With the ability to bind to a myriad of antigens, Ig can recognize antigens and activate cell, while soluble molecules can combine with external substances or pathogens and neutralize pathogens. As a result, Ig is significantly important for physiology and pathology of the human body. The dysregulation of Ig has been identified across different conditions. For example, the structure and composition of IgG or IgM are changed in neurodegenerative diseases such as Parkinson’s disease (PD) or Alzheimer’s disease (AD) (7), while increased IgE is associated with autoimmune diseases such as rheumatoid arthritis (RA) (8). Other type of Ig such as IgA is also involved in autoimmune diseases or centralnervous system diseases. These diseases are commonly thought to be age-related diseases and possess some similar pathological mechanisms with aging.

In this review, we summarize current knowledge on Ig alterations associated with aging and age-related diseases, discuss the underling mechanism of Ig in aging, which will help understanding the mechanism of aging and provide insights into the novel therapies in age-related diseases.

## 2 Immunoglobulins in brief

Ig is a class of proteins existed in gnathostomes such as mammals, birds, and amphibians et al. (9, 10). Although they had already emerged 500 million years ago, its existence was only described in the late 19th century. Ig is critical for protecting pathogen invasion and thought to be one of the hallmarks of adaptive immunity. They are composed of four polypeptide chains: two light chains and two heavy chains according to their molecular weight (about 23.000 and 50000 respectively). Disulfide bonds link the chains and form a Y-shaped structure. These chains contain looped structures and can be divided into different domains or regions. Light chains possess one constant region (CL) and one variable region (VL). The variable region contains three complementary decision regions (CDRs), which contribute to antigen binding. The constant domains specify effector functions including activation of complement or binding to Fc receptors (11). According to the difference in polypeptide sequence, the light chain is classified into kappa (κ) or lambda (λ), which possesses different antigenic properties. The light chains are usually short of carbohydrate components (12). Unlike light chains, heavy chains possess one variable region (VH) and 3-4 constant regions (CH). Based on the size and amino acid composition within constant region, the heavy chains can be classified into different classes. In mammals such as human and mice, five heavy chain isotypes have been identified: α, δ, ϵ, γ, and μ. Therefore, Ig is classified into five isotypes as IgA, IgD, IgE, IgG, and IgM. However, in other species such as bony fish, the heavy chains are μ, δ, and τ/ζ, which represent IgM, IgD and IgT/Z, respectively (13). And in amphibians, the heavy chain isotypes are μ, δ, χ, υ, and C, which represent IgM, IgD, IgX, IgY, and IgF (14).

A significant event called somatic recombination occurs in the V region, which crucially contributes to the diverse repertoire of Ig heavy and light chains. This process includes the recombination of variable (V), diversity (D) and joining (J) gene segments in an ostensibly random manner. Recombination‐activating gene1/2 initiate the V(D)J recombination by introducing double‐strand breaks at specific recombination signal sequences (15). Apart from V(D)J recombination, other factors contributing to Ig function include activation-induced cytidine deaminase-mediated somatic hypermutation (SHM) and class switch recombination (CSR). SHM can induce point mutations in some hotspots and alter the affinity of Ig (16), while CSR leads to the production of secondary isotypes including IgG, IgA and IgE (17). Following these processes, Ig acquires the ability to recognize and bind to a variety of antigens, and finally exert specific effector functions.

Ig is predominantly produced by B cells. Interestingly, macrophages and non-immune cells such as cancer cells and neurons can also produce Ig such as IgG, IgM, and IgA (18). However, their functions remain to be demonstrated.

## 3 Aging affects B cell development and function

**Figure 1:**
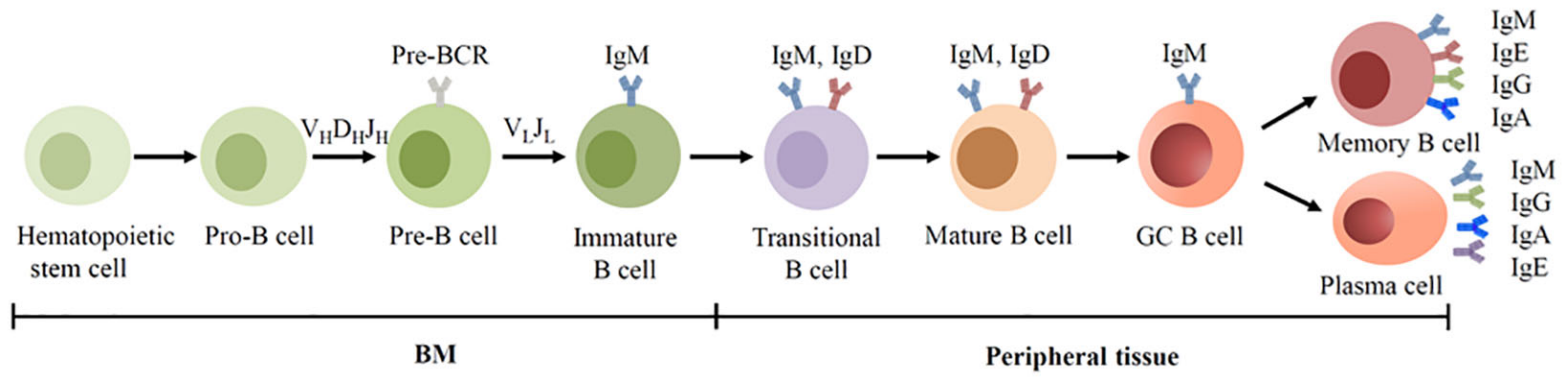
BCR expression during human B cell development. B cells are derived from hematopoietic stem cells in the BM, and acquire BCRs through V(D)J recombination of Ig genes in the early development. In the GC, B cells undergo SHM and CSR, allowing the BCR switched from IgM to other isotypes.

In mammals, Ig is predominantly expressed by B cells which are originated from multipotent hematopoietic stem cells in the bone marrow (BM). Based on the surface marker and the rearrangement of Ig genes, the development of B cells can be categorized into distinct stages: progenitor B cells (pro-B cells), precursor B cells (pre-B cells), immature B cells, transitional B cells, mature B cells, germinal center (GC) B cells, memory B cells and plasma cells (19). Pro-B cells undergo V(D)J recombination and form the μ chains, and develop into pre-B cells. Pre-B cells then form the light chains and develop into IgM^+^ immature B cell (15). Immature B cells subsequently migrate to the spleen for terminal maturation. With the assistance of alternative splicing, immature B cells develop into mature IgM^+^IgD^+^ B cells. Upon antigen encounter, activated B cells differentiate into Ab-secreting plasma cells. The Ab (Ig) secreted by plasma cells shares structural homology with membrane-bound BCR (**Figure 1**).

Two lineages can be divided in the early development of B cells, B1 and B2. B1 cells are originated from fetal liver progenitor cells, and notably, their BCR lacks nucleotides at the junctions between V and J segments (20). Located in the peritoneal cavity, most B1 cells can produce natural antibodies including IgM, IgA and IgG in humans and mice. Unlike conventional B2 cell-produced adaptive Ab, natural Ab is synthesized without exposure to foreign antigen. They are polyreactive and can recognize numerous autoantigens and new antigens (21, 22). Conventional B2 cells are mainly located in peripheral tissues and consist of two main populations: marginal zone (MZ) B cells that rapidly produce IgM upon antigen exposure and follicular (FO) B cells produce high-affinity Ab such as IgG, IgA, or IgE.

The emergence of autoantibody (autoAb) manifests a functional collapse of B cell tolerance. B cell acquires central and peripheral tolerance during development. Central tolerance is achieved by the rearrangement of Ig chains and subsequent BCR formation. This process can form autoreactive B cells. Upon reacting with autoantigens in the BM, these newly developed B cells undergo negative selection, a process results in the elimination of some autoreactive immature B cells. The surviving cells then immigrate to the periphery and undergo periphery tolerance, which further eliminate autoreactive cells. The breakdown of both central and peripheral tolerance contributes to the increase of autoreactive B cells, and thereby increased circulating autoantibodies.

Apart from B cells, other cells are involved in the production of autoAb in some conditions. T cells can regulate B cell function. The dysregulation of follicular helper T cells affects B cell maturation and promote the production of autoAb (23). Macrophages play important roles in antigen presentation, immune tolerance and inflammatory response. With increased antigen presentation, macrophage can help to activate B cells and boost the production of autoAb. Moreover, elevated apoptotic debris of macrophage is also involved in autoAb formation in autoimmune diseases (24, 25). Increasing studies have proved that autoAb exert both harmful and protective effect. On the one hand, autoAb is the major contributor in initiating autoimmune diseases. On the other hand, autoAb can suppress inflammation, infection, and kill tumor cells in various diseases (26).

**Figure 2:**
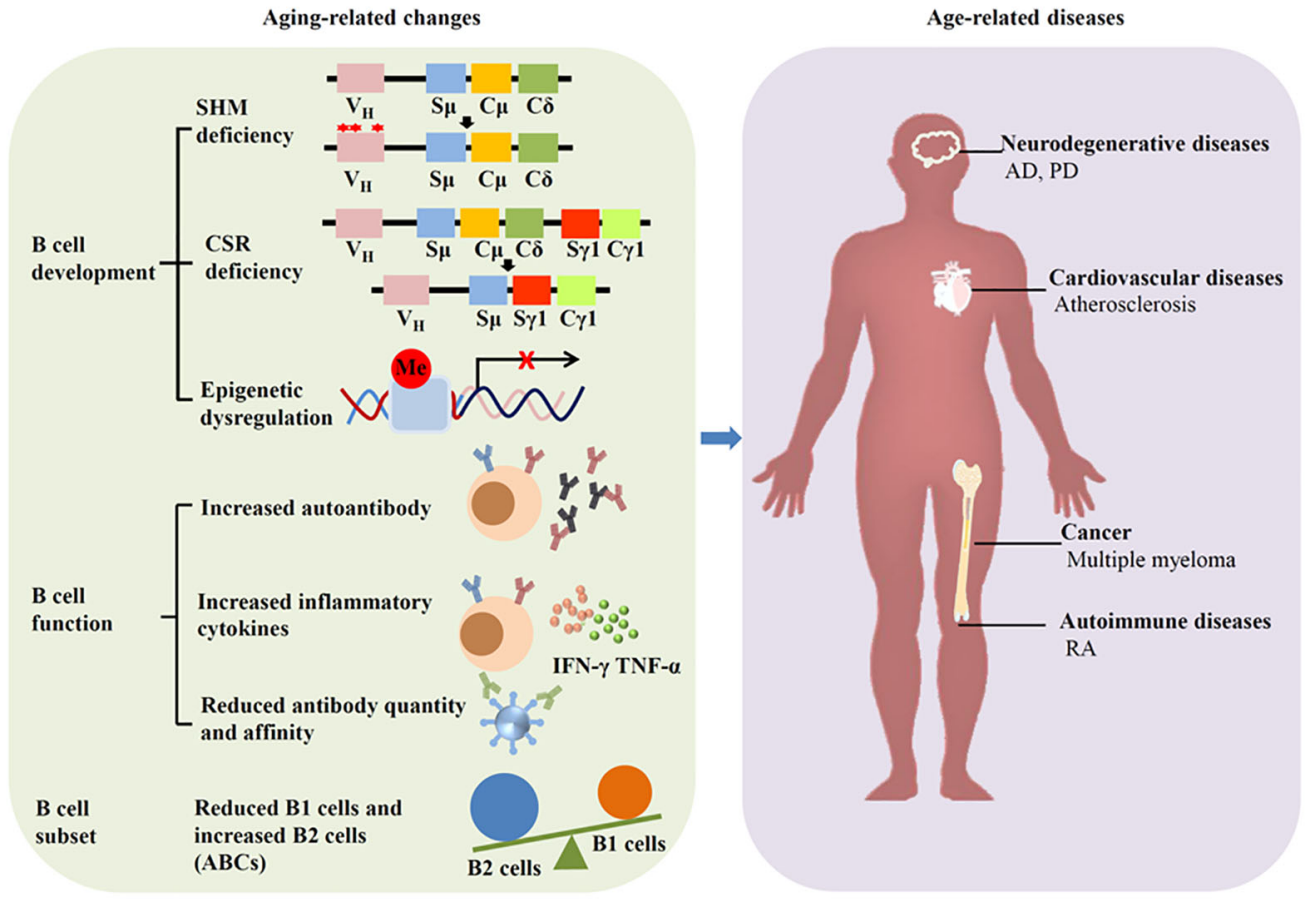
Aging affects B cells and induces diseases. Aging can alter the development, function and subset of B cells, leading to age-related diseases such as neurodegenerative diseases, cardiovascular diseases, autoimmune diseases, and cancer.

Aging induces great changes in immune system, especially in humoral immune mediated by B cells. This phenomenon is called immunosenescence. Immunosenescence can increase both the frequency and severity of infectious diseases, and reduce the response to vaccination. In fact, aging can affect the development and subset of B cells, as well as Ab production (**Figure 2**).

During aging, murine and human BM demonstrates diminished B cell precursors, likely attributable to age-associated microenvironmental changes. While murine B1 cell numbers remain stable with aging (27), human B 1 cells are reduced in the elderly (28). In accordance with reduce B1 cells, the amount and function of natural antibodies is reduced during aging, leading to age-related diseases such as atherosclerosis, cancer, and neurodegeneration (29–31). B2 cells exhibit aging-related functional impairments, as manifested by defective isotype switching in aged cells (32). Although peripheral B2 cell maintain unchanged in aging mice, the population of FO B cells decreases with age. The amount of MZ B cell is also reduced during aging, which accompanied by the augmentation of autoantibodies (33, 34).

Age-associated B cells (ABCs) are presumably derived from B2 cells and represent a memory B cells. They are accumulated in the spleen of aged mice. In humans, ABCs expand in elderly individuals and correlate with increased IgG1 levels. ABCs demonstrate autoreactive potential through autoAb secretion.

AutoAb is not only related to autoimmune disease, but also is associated with aging. In fact, there is a complex relationship between aging and autoAb. Increased autoAb is identified during aging. Among them, antinuclear Ab is related to diabetes and age-related diseases (35). Autoantibodies against AT1 receptor can promote endothelial cell senescence and vascular aging (36). Aging can alter the inflammation response as evidenced by increased proinflammatory cytokines such as TNF-α and IFN-**γ**. Chronic low-grade inflammation, as we all know, is a characteristic of aging.

## 4 Immunoglobulins and aging

### 4.1 Immunoglobulin level changed with advanced age

Aging significantly influences B cells, which can be reflected by the changes of Ig levels. Although serum IgE remains stable, the concentrations of switched Ig (IgA and IgG) exhibit increased expression in the human elderly, while IgM and IgD are reduced with age (37). This change implies a transition from naïve to memory B cells and impaired response to new antigens (38). Interestingly, the level of total serum Ig shows no difference or a mild elevation with advanced age in human (39, 40). In addition, the glycosylation of Ig can also be altered with age, which plays an important role in aging and age-related diseases (**Table 1**). The following sections will review age-related changes in different Ig classes, and go a step further to discuss the underlining mechanism, which will be helpful in understanding the complexity of Ig in aging.

**Table 1:** Aging-related changes of immunoglobulin.

Immunoglobulin	Serum concentration	Glycosylation	References
IgM	Reduced in human Increased in mice	/	(37, 49)
IgG	Increased in human and mice	Reduced galactosylation and sialylation in human, Increased agalactosylation in human	(37, 64, 70)
IgA	Increased in human and mice	Reduced galactosylation and sialylation in human, Increased bisection in human	(37, 102)
IgD	Reduced in human	/	(37)
IgE	No change in human	/	(37)

### 4.2 IgM and aging

#### 4.2.1 IgM structure and function

IgM is the predominant natural Ab and serves as the first responder to foreign invaders. It is the only Ab found in all vertebrate species. Monomeric IgM mainly exists as membrane-bound receptors on B cells, playing a vital role in B cell survival (41, 42). The monomeric IgM has two μ-heavy chains with a C-terminal extension that facilitates oligomerization. Under certain pathological condition such as autoimmune diseases, monomeric IgM can also be secreted (43, 44).

As the primordial class of Ab produced by activated B cells, IgM typically forms a pentameric structure upon secretion. Five IgM monomers are joined together by disulfide bonds and form this pentamer with the assistance of J-chain. The presence of the J-chain enables IgM to cross mucosal epithelia through interaction with the polymeric Ig receptor (45). The pentameric configuration enhances IgM’s ability to bind antigens with high avidity, allowing it to perform multiple functions in immune responses (46).

While human and mouse IgM primarily exist as pentamers, other forms, such as hexameric IgM lacking the J-chain, have been observed in frog. IgM plays a significant role in both humoral and mucosal immunity. It is highly effective in recruiting complement and inducing strong inflammatory responses. Additionally, pentameric autoreactive IgM has been implicated in various autoimmune diseases, including RA and autoimmune neuropathy (47).

#### 4.2.2 IgM exerts a complex role in aging

Aging can increase the level of IgM in mice. Aged mice express more serum IgM than young mice. In response to *S. aureus* bacteremia infection, aged mice demonstrate higher IgM levels compared to their young counterparts (48–50). However, the situation is different in human. Human serum IgM was reduced with age, especially in women (37). A possible reason for the difference between humans and mice is related to the dynamic changes in B1 cells. B1 cells are mainly producers of IgM. Human B1 cells reduce with age, while mice B1 cells do not. Chinese centenarians with lower serum IgM levels had significantly shorter median survival time (51). These findings suggest that IgM may provide protective effects in human elders.

Time-restricted eating (TRE) is an intermittent fasting pattern that limits daily eating time to a window ranging from 4 to 12 hours. TRE possesses the anti-aging ability as evidenced by increased sphingosine-1-phosphate and L-serine expression. The percentage of IgM increased after 30 days of TRE. The activation of B cells is suppressed as demonstrated by reduced CSR from IgM to IgA (52). Despite the lack of direct evidence, this study indicates that IgM may possess potential anti-aging capability. The protein in cerebrospinal fluid (CSF) is closely associated with aging and neurodegenerative diseases. Using limited proteolysis-mass spectrometry, researchers find that the structure and expression level of IgM are changed in human CSF with aging (53). To be more specific, a complex composed of IgM and Cd5l is increased in mouse and human CSF with aging, which may provide protection against PD. Adaptive immunity is involved in the development of atherosclerosis. IgM can reduce atherosclerosis progression and cardiovascular events (54–56). Patients with coronary artery disease exhibit a significant decrease in circulating atheroprotective oxLDL-specific IgM compared to young healthy volunteers (57). Systemic lupus erythematosus (SLE) patients always show increased atherosclerosis. Serum IgM antibodies against phosphorylcholine (anti-PC), which can provide protection against atherosclerosis, are reduced with age in SLE patient. The protective role of IgM anti-PC antibodies may be associated with the senescence of T cells (58, 59). Based on these findings, it is suggested that IgM can be protective in aging and age-related diseases.

Notably, some investigations yield inconsistent results. Increased urine IgM is linked to the development of vascular aging and cardiovascular events (60). Vascular aging is evidenced by the changes of vascular structure and function, and plays a crucial role in brain and cognitive aging. Many factors such as arteriosclerosis or endothelial dysfunction emerged as the early stage of vascular aging (61). In young to middle-aged healthy people, the increased IgM expression in urine is not associated with hypertension but often means lower ankle brachial index and higher systolic blood pressure. Most importantly, elevated urinary IgM is closely related to increased urinary albumin excretion, an indicator of systemic inflammation and cardiovascular abnormalities. Based on these findings, urine IgM can be seen as an indicator of subclinical peripheral atherosclerosis (62).

### 4.3 IgG and aging

#### 4.3.1 IgG structure and function

IgG is the predominant Ig class in healthy humans (about 80% of total serum Ig). Structurally, IgG is a 150 kDa glycoprotein composed of two identical heavy chains and two light chains. The heavy chain contains one VH and three CH (CH1, CH2, and CH3). The hinge region exists between the CH1 and CH2 region and contribute to conformational flexibility of the IgG molecule. The protease papain cleaves the hinge region at the N-terminal side of the disulfide bonds and split IgG into three pieces: two identical Fab fragments (fragment antigen binding) and one Fc fragment (fragment crystallizable). The Fab fragments are capable of recognizing and binding to a variety of antigens including bacteria, toxins or self-antigens. The Fc fragment interacts with specific receptors on the surface of immune cells and induce immune responses (49). Pepsin cleaves IgG molecule at the C-terminal of the hinge region and then produces an F(ab’)2 fragment and a smaller Fc fragments (pFc’) (63). Based on the heavy chain γ1-4, IgG can be divided into four subclasses: IgG1, IgG2, IgG3, and IgG4. Despite the sequence homology, these subclasses show subtle differences in conformational flexibility or binding affinity which affect their function.

IgG can cross the placenta and diffuse into extravascular areas. It is critical to humoral immune as protecting against pathogens. IgG exert their protective function through binding to Fcγ receptors (FcγR) and then activating FcγR-bearing cells. IgG can also activate complement which contribute to the recruitment of immune cells.

#### 4.3.2 The reciprocal interaction between IgG and aging

**Figure 3:**
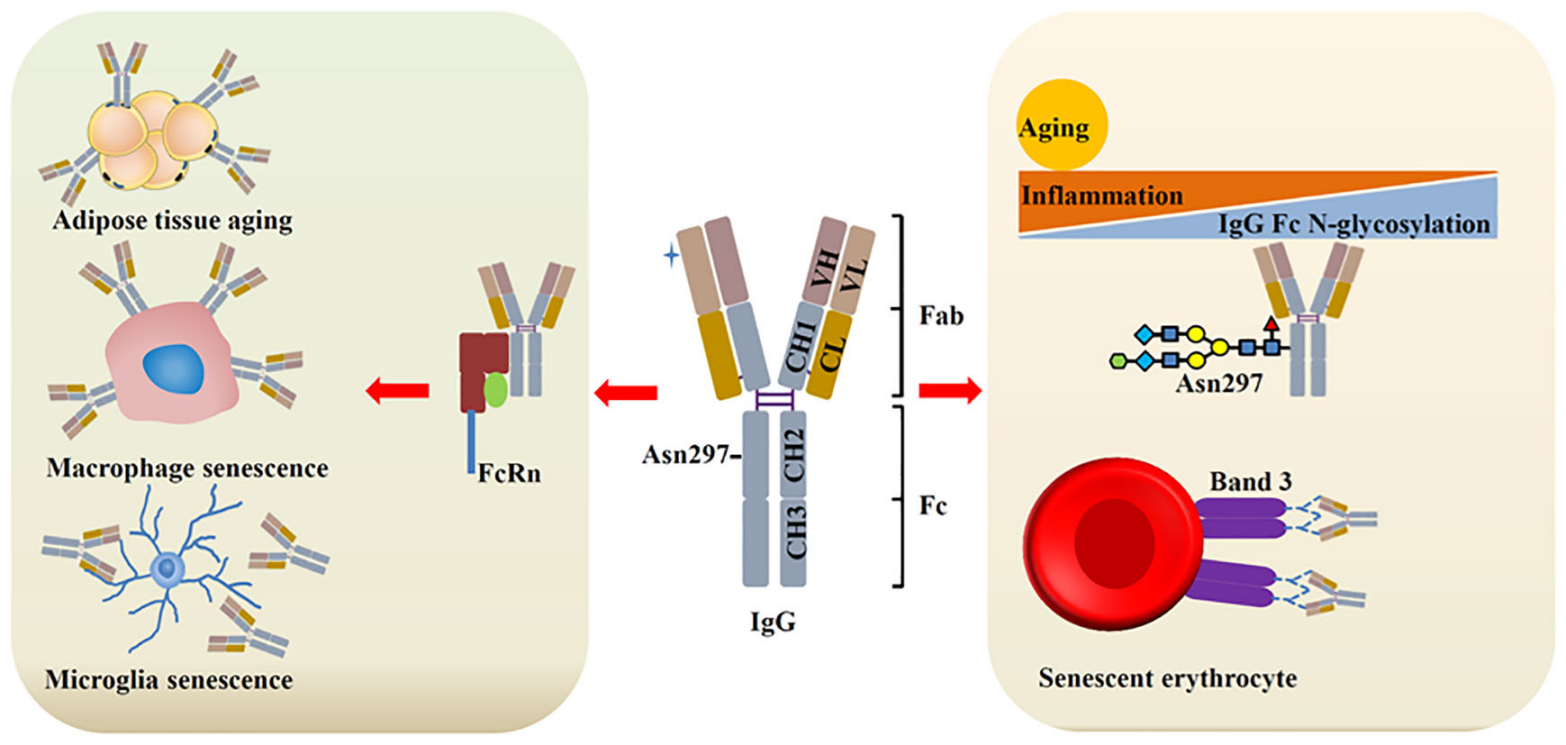
IgG and aging. During aging, IgG is increased, which contributes to cell senescence and tissue aging through binding with different ligands. Aging/inflammation can reduce the N-glycosylation (galactosylation and sialylation) of IgG, leading to increased inflammation, which further promote aging. Senescent erythrocytes show increased IgG binding with band 3 protein.

IgG is the most extensively studied aging-related Ig to date. The latest research indicates that the accumulation of IgG is a hallmark of human aging. IgG is increased in human liver and lymph node with age. Moreover, accumulated IgG can induce cell senescence and contributes to tissue aging (3). IgG-producing cells are increased in aged mice, which are consistent with the levels of serum IgG (64, 65). However, in SAMP1 mice, serum IgG was reduced sharply, which contribute to aging-associated arterial stiffening. IgG treatment alleviates arterial stiffening and hypertension in old mice (66). Although the results may vary, these findings all demonstrate this idea: Aging can alter the expression and function of IgG, and IgG can in turn affect aging. Regulation of IgG can counteract aging (**Figure 3**).

#### 4.3.3 IgG N-glycosylation and aging

Glycosylation is the most frequent post-translational modification. It mediates cell adhesion, proliferation and differentiation. N-linked and O-linked glycosylation are the two main forms of glycosylation. The function of IgG is modulated through N-glycosylation, where glycans attach to Asn297 in the consensus sequence (Asn-X-Ser/Thr). Under homeostatic conditions, N-glycosylation of IgG remains stable. However, inflammation and aging can significantly alter IgG glycosylation. As a result, changes in IgG glycosylation are closely associated with aging and age-related diseases (67). N-glycosylation includes galactosylation, sialylation, core-fucosylation, and bisecting GlcNAcylation (68). Of which, core-fucosylation and bisecting GlcNAcylation exhibit slightly alteration with age. On the contrary, the level of galactosylated IgG glycans is increased from young and reduced with advancing age. IgG glycosylation can be a predictor of human aging (69, 70). In mice, serum IgG N-glycans also changed with aging. B-cell-specific ablation of β-1,4-galactosyltransferase1can maintain IgG glycans and attenuate aging in mice (71). Aberrant glycosylation of IgG is closely linked to age-related diseases, including dementia, hypertension, and diabetes. Chronic inflammation is crucial for aging and is a causal factor in age-related diseases (72, 73). IgG galactosylation can be reduced by chronic low-grade inflammation, and the attenuation of IgG galactosylation further promotes inflammation. A mendelian randomization study proved the potential causality between IgG N-glycosylation and aging, sialylation of IgG can reduce the inflammation and suppress the aging process (74). Although the mechanism by which glycosylated IgG regulation inflammation remains unclear, the latest study has identified a key transcription factor: repressor element-1 silencing transcription factor (REST). Sialylated IgG can activate the REST in macrophages, which can suppress nuclear factor κB-related signals, leading to reduced inflammation (75).

#### 4.3.4 IgG and erythrocyte aging

Erythrocyte is originated from hematopoietic stem cell and shows a short lifespan of 115 days in the blood. Erythrocyte level is declined in the elderly. Band 3 protein is predominant expressed on erythrocyte membranes and plays an important role in erythrocytes homeostasis. Recent studies suggest that band 3 protein is important for erythrocyte senescence signaling (76). Band 3 anion exchanger can produce senescent cell antigen that binds to serum IgG. This process is important for the removal of erythrocytes (77, 78). Senescent erythrocyte binds with more IgG than young erythrocyte. In neurodegenerative diseases such as AD and PD, band 3 exhibits increased degradation and produce more senescent cell antigen, which may finally contribute to increased IgG on the surface of erythrocytes (79). Erythrocyte can be seen as a biomarker of AD, which indirectly indicates the involvement of IgG in neurodegenerative diseases. These findings are consistent with recent study that IgG is highly expressed near the senescent cell and promote senescence.

#### 4.3.5 Tissue IgG and aging

IgG is accumulated in white adipose tissue and induces the degeneration of adipose tissue during aging (74, 80), which suggests that IgG not only exerts immune function in the plasma, but also plays an important role in metabolism in the tissue. In addition of adipose tissue, IgG has been found accumulated in different tissues and induce the senescence of nearby cells (53). The accumulated IgG is regulated by FcRn. FcRn can protect IgG from lysosomal degradation upon binding with it. Targeting FcRn can reduce IgG expression and aging. Bidirectional two-sample mendelian randomization analysis identifies the causal association with SASP (81). Another report finds that IgG is accumulated in the tissues of aged mice and elicit the senescence of macrophage and microglia, which further aggravates aging. Reducing IgG can attenuate aging.

### 4.4 IgA and aging

#### 4.4.1 IgA structure and function

IgA serves as the predominant Ab class in humans, constituting approximately 3/4 of total Ig. Although IgA is mostly distributed in mucosal tissues, it ranks as the second most abundant plasma Ig following IgG. Based on the molecular forms, IgA exists in two distinct forms: monomeric IgA (mIgA) and polymeric IgA (pIgA). Plasma IgA exists mainly in a monomeric form. On the contrary, over 90% of mucosal IgA displays polymeric forms.

The human IgA comprises two subclasses (IgA1 and IgA2) distinguished by amino acid sequences of the hinge region. Both subclasses associate with either κ or λ light chain consisting of VL and CL domains. The heavy chains contain a VH followed by three constant domains (Cα1-Cα3). The ratio of IgA1 to IgA2 in serum is 9:1. IgA2 demonstrates two principal allotypes IgA2m (1) and IgA2m (2). A third variant, IgA2 (n) has been recently characterized. This subclass diversity contrasts with mouse or rat where a single IgA form predominates.

Functionally, IgA serves as the primary immunological barrier at mucosal surfaces. Its functional repertoire includes neutralization, complement activation, maintenance of host-commensal homeostasis, and receptor-mediated effector functions. Interestingly, the functions of IgA can be performed by other Ig such as IgM, IgD, or IgG. In fact, IgM is similar to IgA in many aspects such as evolution, structure, and function (42).

#### 4.4.2 IgA interacts with microbiota during aging

IgA is responsive to age and show significant changes with aging. Human serum IgA is increased during aging, which is similar to mice (82). Older people have more IgA in the urine (83). Blood plasma therapy can decrease IgA expression in old rat (84). Moderate aerobic exercise can promote IgA production and improve homeostatic conditions during aging (85). CCL25 is a chemokine that recruits IgA-secreting cells into intestinal lamina propria. Aging can reduce the expression of CCL25. This reduction can in turn decrease IgA and IgA-secreting cells, ultimately affecting gut immunity (86, 87).

A notable phenomenon is that IgA, gut microbe and aging are interrelated and mutually influential. IgA is produced into the intestinal lumen in large numbers every day. These secreted IgA can interact with gut microbiome, and then maintain host-microbiota homeostasis (88, 89). IgA can change the bacterial composition and inflammatory response in the intestinal tract. Gut microbiota dysbiosis is detected in IgA deficient human (90). As a result, IgA is accepted as a controller of symbiotic microbiota (91). Studies have shown the composition of human gut microbiome changed during aging. Microbiota with beneficial functions (such as *Oscillospira*, *Oxalobacter*, *Prevotellaceae*) declined with age, while others (*Parvimonas*, *Corynebacterium*, and *Corynebacterium*) increased and are associated with aging-related inflammation and diseases (92–95).

Cellular senescence is a hallmark of aging. IgA seemed to serve as a bridge connecting the gut microbiota and cellular senescence during aging (96–98). In the ileal of aged mice, commensal bacteria may promote the senescence of GC B cells. The accumulated senescent cells lead to compromised diversity and production of IgA, which in turn changes the composition of gut microbiota and break the gut homeostasis.

It is noteworthy that gut microbiome not only influences IgA that is in the same location, but also exerts an influence on IgA secretion that is anatomically distant from gut. Endocrine dysfunction is associated with gut microbiome during aging. The pituitary gland is an important endocrine organ. During aging, pituitary hormone declined. Interestingly, IgA can be produced by hormone-secreting cells but not B cells in pituitary. The expression of IgA is increased significantly in aged pituitary, which is regulated by gut microbiota (99).

Gut microbiota can also regulate IgA in the central nervous system. IgA^+^ B cells are increased in the CSF of MS patients. These cells are related to the acute inflammation and neuroinflammatory conditions. Gut microbiota-specific IgA may transported to the CNS and induce neuroinflammatory diseases (100). Moreover, IgA can affect microbiota and further influence lymphocyte and glial cells in the central nervous system (101). Aging-related reduction of microbiota composition can reduce the maturation of microglia. Although microbio is important in CNS aging and IgA can regulate microbio, there is no direct evidence to support the idea that IgA can regulate CNS aging through microbe at present. The interplay among IgA, the microbiome and aging-related CNS disease require further investigation.

#### 4.4.3 IgA glycosylation and aging

Like IgG, IgA contains abundant N- and O-glycosylation sites. These glycopeptide structures changed during aging. Notably, the glycomes of IgA and IgG are closely correlated and regulated by common genetic factors (102). While IgG glycosylation is important in aging, the role of IgA glycosylation in aging remains unknown. Given IgG’s established role in aging, the relationship between IgA glycosylation and aging is worthy of further exploration.

### 4.5 IgD and aging

#### 4.5.1 IgD structure and function

IgD has two delta (δ) heavy chains which is different from IgG, IgA, and IgM. It exists in all vertebrate species and thought to be evolutionarily conserved Ig class. IgD exhibits pronounced structure plasticity in different species, probably through extensive modifications via both the duplication and deletion of exons. For example, mice and human IgD consisted of two and three Cδ domains, while catfish IgD has seven Cδ domains. Moreover, most jawed vertebrate species display significant alternative RNA splicing events compared to mammals, which also involved in the structural plasticity of IgD.

In mammals such as human and mice, IgD exists in two forms: membrane-anchored IgD (mIgD) and secreted IgD (sIgD). Expression of mIgD follows IgM during B cell development and is an important component of BCR. Most mature B cells coexpress surface IgD and IgM through alternative splicing of a pre-messenger RNA in the nucleus. Notably, a unique subset of mucosal B cells in nasopharyngeal lymphoid tissues exhibit exclusive IgD expression through an unconventional CSR mechanism. These IgM^−^IgD^+^ B cells then differentiate into IgD-secreting plasma cells (103, 104). sIgD remains minimally detected in the serum, which accounts for only 0.25% of total serum Ig (105). However, it demonstrates broad tissue distribution, detectable in the nasopharyngeal, oral and lachrymal secretions. Moreover, sIgD can traverse epithelial and placental barriers (106). The distribution of sIgD is correlated with the localization of IgD-producing B cells.

The functions of IgD are relatively enigmatic compared to other Ig. mIgD on B cells may be involved in the peripheral tolerance and B cell anergy, while sIgD may help to maintain mucosal homeostasis through regulating symbiotic host-microbiota interaction (107, 108). The relationship between IgD and aging is also unknown, with only indirect insights from the studies on B cells.

#### 4.5.2 IgD^−^CD27^−^ B cells and aging

IgD is an important marker of B cells. According to the expression of IgD and/or CD27, human B cells can be classified into four subsets: naïve B cells (CD27^-^IgD^+^), unswitched memory B cells (CD27^+^IgD^+^), switched memory B cells (CD27^+^IgD^−^) and double‐negative (DN) B cells (CD27^−^IgD^−^) (109). CD27^−^IgD^−^ DN B cells are also thought as a subset of memory B cells (110, 111). IgD^+^IgM^+^CD27^+^ memory B cells are dramatically declined in the aged people (112). Although circulating IgD^−^CD27^−^ B cells exhibit lower expression of SASP marker including TNF-α, IL-6, IL-8 and p16INK4 (113), they are now thought to be related to immunosenescence, aging, autoimmune and infectious diseases. It is increased in elder people and had been seen as senescent or exhausted B cells. In HIV patients, the number of IgD^−^CD27^−^ cells is correlated with CD3^+^CD4^+^CD57^+^CD45RO^−^CD4^+^ T cells, a terminal effector cells that are prevail in aging (114). In addition, IgG^+^IgD^−^CD27^−^ B cells are increased in RA patients. IL-6R blockade (tocilizumab) or TNF inhibitors can significantly reduce the expression of cells to normal levels (115, 116). Moreover, IgD^−^CD27^−^ B cells are associated with severe atherosclerosis in human and can promote inflammation in male elders (117, 118). All these findings suggest that IgD^−^CD27^−^ B cells may play a role in aging-related inflammation or diseases.

### 4.6 IgE and aging

#### 4.6.1 IgE structure and function

Amphibians IgY underwent a gene duplication event and diverged into IgE, a unique Ab class which is exclusively found in mammals. IgE exists as a monomeric form composed of two heavy and two light chains, distinguished from other Ig isotypes by its characteristic epsilon constant region (Cϵ). The Cϵ region contains four constant domains (Cϵ1-Cϵ4), which is similar to the μ heavy chain of IgM. Despite sharing evolutionary origins with IgG through ancestral IgY molecules, IgE exhibits distinct structural features, particularly in its Cϵ2 domain positioning. These domains occupy spatial coordinates equivalent to the Fab-Fc hinge region found in IgG molecules, a key differentiating feature between these two Ab classes. Cϵ2 and Cϵ3 domains interact with the high‐affinity IgE‐receptor FcϵRI, while Cϵ3 and Cϵ4 domains interact with the low‐affinity IgE‐receptor CD23. CD23 on B cells is important for IgE synthesis and presentation.

IgE demonstrates the lowest serum concentration among Igs (approximately 10,000 fold less than other isotypes). However, it exhibits remarkable potency in host defense against parasites and certain toxins. This Ab class plays a dual biological role, mediating both protective immune responses and pathological hypersensitivity reactions. Membrane-bound IgE antibodies undergo crosslinking upon encounter antigens, and then initiate the release of bioactive molecules collectively induce allergic and inflammatory reactions.

#### 4.6.2 IgE is involved in age-related diseases

Although previous studies showed that IgE production is reduced with age and is associated with reduced allergic symptom (119), recent findings require a fresh look at the role of IgE in aging. IgE is closely associated to allergy, a disease which is considered a pediatric disease rather than an adult one. Interestingly, some studies find that allergy, especially food allergy is increasing in elders, which is similar to other age-related diseases such as cardiovascular, neurodegenerative, and cancer (120, 121). Many factors seemed to be involved in this phenomenon. Immunosenescence play a crucial role in food allergy. Food allergy indicates the impairment of mucosal tolerance. Gut immune system experiences multiple changes during aging, such as increased local inflammation, impaired barrier function, and IgA deficiency. Aging also alters the composition and function of gut microbiota, leading to chronic inflammation. IgE is increased in the skin lesion of elders, which can induce inflammatory response and contribute to allergy in elders (122). In addition, B cells are previously thought to be cleared shortly after IgE production. However, long-lived B cells that constantly producing IgE have been identified, supporting the increased IgE with aging.

IgE also contributes to autoimmune diseases. Autoreactive IgE can activate basophils, and other FcϵRI-bearing cells, prompting Ab production and other pro-inflammatory signals. IgE autoantibodies have been identified in rheumatic diseases such as RA and SLE (8). Therapy targeting IgE can reduce autoimmune diseases. In addition, serum IgE is increased in atherosclerosis patients. This may attribute to IL-17, an important proinflammatory cytokine in the pathogenesis of atherosclerosis. IL-17 is increased in atherosclerosis, which can enhance B cell-produced IgE. IgE can promote macrophage polarization and cholesterol accumulation through binding with FcϵR1. IgE deficient mice demonstrate attenuated atherosclerosis (123, 124).

## 5 Therapeutic potential of Ig in aging

**Figure 4:**
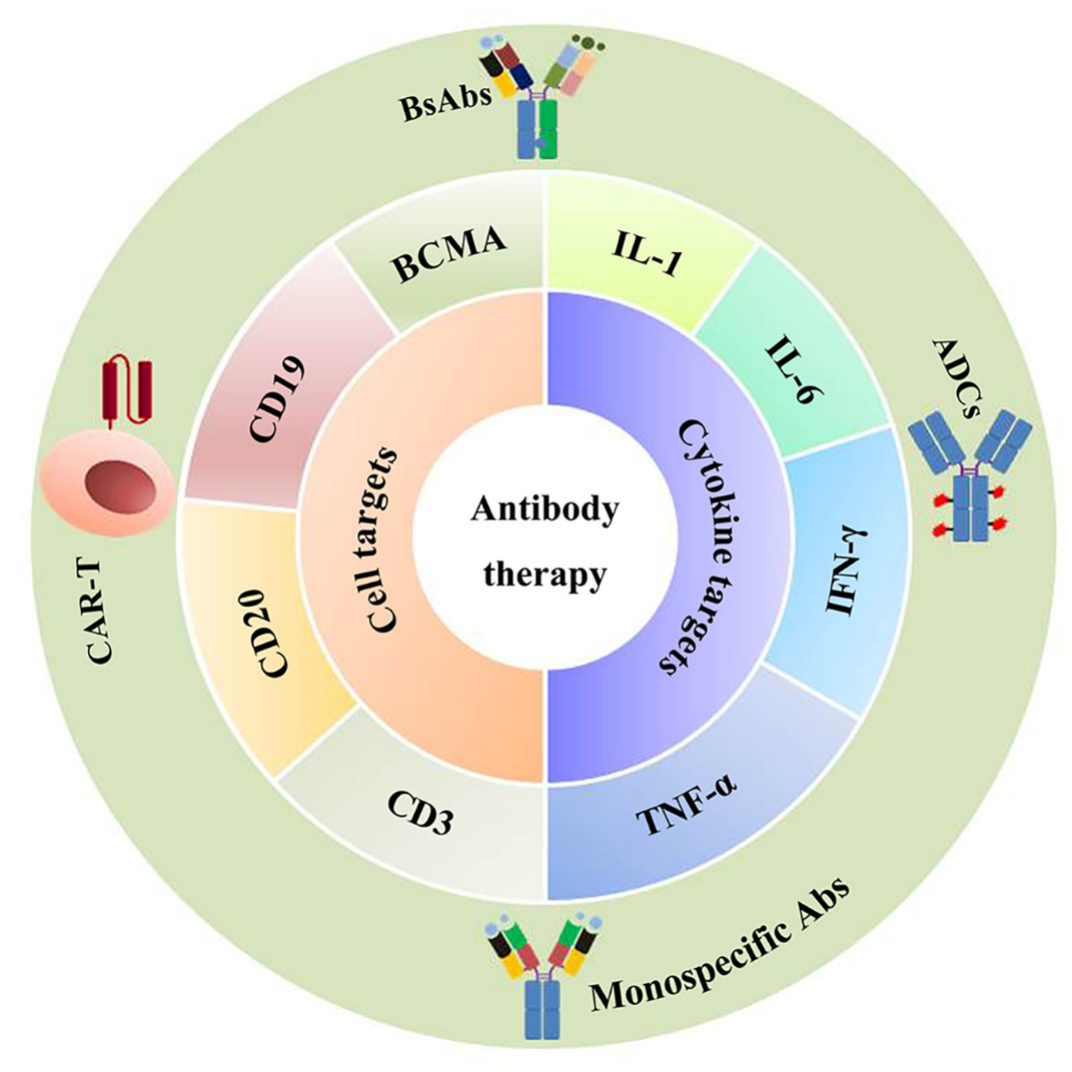
Antibody-based therapy. Antibody-based therapy is used to treat age-related diseases such as cancer or autoimmune diseases through regulating inflammation or senescence. The currently developed drugs include monospecific Abs, bsAbs (bispecific Abs), CAR-T (chimeric antigen receptor T), and ADCs (antibody-drug conjugates), which target CD19, CD20, BCMA (B cell maturation antigen), or target inflammatory cytokines such as IL-1, IL-6, TNF-α, and IFN-γ.

Ig therapy exerts anti-inflammation and immune modulatory function, and has been used in immune deficient diseases and other immune-mediated conditions such as dermatomyositis, autoimmune bullous diseases, and chronic inflammatory demyelinating polyradiculoneuropathy (125). However, the therapeutic effect in aging remains to be clarified. Recent studies find that antisense oligonucleotide against FcRn can inhibit the accumulation of IgG in adipose tissue, which helps reduce fibrosis and relieve aging (74, 80). In another study, suppressing IgG effectively reduces aging in mice. IgM transfer has been reported to induce B cell tolerance and inhibit autoimmunity (126). Considering the fact that Ig is deeply involved in the development of aging, there is a high possibility that regulation of Ig can be used to develop novel methods for counteracting aging. Chronic inflammation or inflammaging is the hallmark of immunosenescence and aging, and anti-inflammatory therapies can be seen as potential anti-aging treatment (127, 128). B cells are the main Ig-producing cells and are critical in immune response. Interventions targeting B cells may alleviate aging through regulating inflammation, senescence and Ig production. Rituximab, a monospecific Ab that targets CD20 on B cells, can alleviate RA, MS, and atherosclerosis. However, monospecific Ab only targets one specific molecule, leading to limited efficacy. Different approaches have been developed to improve therapeutic efficacy. Bispecific Ab (BsAb) can bind two different epitopes/antigens simultaneously, which aids in redirecting cytotoxic effector cells to target cells. Ab-drug conjugate (ADC) consists of a monoclonal Ab and a cytotoxic drug. This combination helps to achieve targeted and potent therapy. Besides bsAb and ADC, chimeric antigen receptor T (CAR-T) cell therapy also makes encouraging progress in B cell malignancies. It uses engineered T cells to target CD19 and B cell maturation antigen (BCMA). In summary, these new technologies offer considerable potential for therapeutic use and greatly enhance treatment efficacy in autoimmune diseases and cancer (129) (**Figure 4**).

## 6 Conclusions

Aging is an interconnected process during which immune responses experience a gradual decline. Increasing studies suggest that aging can regulate the production and function of Ig through immunosenescence, chronic inflammation, epigenetic modification, and microbe disturbances. The immunosenescent B cells are deficient in Ig class switch and affinity maturation which affect the isotype and function of Ig. On the other hand, with increased inflammatory factor production and self-tolerance broken, aged B cells can produce autoAb which contributes to some age-related diseases. Glycosylation or Asp isomerization in Ig, especially in IgG, has changed during aging and is expected to be seen as a biomarker of aging. In addition, there is a complex relation between aging, microbes, and Ig. Ig and aging exhibit mutual influence and interaction, although the underling mechanisms require further clarification. Moreover, apart from B cells, Ig is produced by many other cells such macrophage. Macrophage is related to aging. However, the function of macrophage-derived Ig remains unknown. Overall, aging is related to almost all chronic diseases. Understanding the role of Ig in aging will facilitate the diagnosis of these diseases. Ig or Ig-producing cells-based therapy may be a hopeful strategy to intervene in aging and age-related diseases.
